# Efficacy of D-mannose as prophylaxis of recurrent urinary tract infection: a systematic review and meta-analysis of randomized controlled trials

**DOI:** 10.1590/2175-8239-JBN-2025-0169en

**Published:** 2025-09-26

**Authors:** Carlos Eduardo Franca Vargas, Antonio Mutarelli, Luis Gustavo Menegardo, Acza Kalica Buarque da Silva, Patricia Rocha Barros Vieira, Jiandra da Luz, Nicole Felix, Luis Claudio Santos Pinto

**Affiliations:** 1 Universidade Federal de Minas Gerais, Faculdade de Medicina, Belo Horizonte, MG, Brazil.; 2 Massachusetts General Hospital, Cardiovascular Research Center, Boston, USA.; 3 Escola Superior de Ciência da Santa Casa de Misericórdia de Vitória, Vitória, ES, Brazil.; 4 Universidade Federal de Alagoas, Faculdade de Medicina, Maceió, AL, Brazil.; 5 Universidade Estadual de Campinas, Campinas, SP, Brazil.; 6 Universidad Nacional de La Plata, La Plata, Argentina.; 7 Universidade Federal de Campina Grande, Campina Grande, PB, Brazil.; 8 Centro Universitário Metropolitano da Amazônia, Belém, PA, Brazil.

**Keywords:** Mannose, Antibiotic Prophylaxis, Urinary Tract Infections, Systematic Review, Meta-Analysis

## Abstract

**Introduction:**

Recurrent urinary tract infections (UTIs) significantly impact the quality of life due to symptoms, effects on sexual activity, persistent pain, and recurrent antibiotic use. This systematic review and meta-analysis aimed to evaluate the efficacy of D-mannose in preventing recurrent UTIs.

**Methods:**

In May 2024, we systematically searched PubMed, EMBASE, and the Cochrane Library for randomized controlled trials (RCTs) comparing D-mannose treatment with no intervention or standard antibiotic therapy in patients at high risk for recurrent UTI. We applied a random-effects model to pool relative risks (RR) and 95% confidence intervals (CI).

**Results:**

We included 6 RCTs comprising 1,167 participants, of whom 534 received D-mannose and 521 (97.6%) were women. D-mannose was not associated with a reduction in recurrent UTI compared with control (RR: 0.57, 95% CI 0.29 – 1.15; p < 0.01) or antibiotics (RR: 0.39, 95% CI 0.12 – 1.25; p < 0.01). Further analyses showed that D-mannose did not improve outcomes in a subgroup of postmenopausal women.

**Conclusion:**

In this meta-analysis of RCTs, D-mannose did not reduce the incidence of recurrent UTIs compared with control or antibiotics in high-risk patients.

## Introduction

Over 400 million patients had urinary tract infections (UTIs) in 2019, and more than 200 thousand died from the condition^1^. Most patients are women and often have recurrent UTIs, with more than two episodes in six months or three in one year^2^. UTIs have a negative impact on quality of life, and can lead to pain and avoidance of sexual relationships.

While antibiotics remain the mainstay treatment, their effectiveness can vary, and long-term use raises concerns about resistance and side effects^2^. Some women benefit from empathetic and knowledgeable healthcare support, which can help alleviate symptoms, but many continue to struggle despite medical interventions^3^. This highlights the need for broader research into alternative treatments, including non-antibiotic options like probiotics or natural remedies, and a deeper understanding of the personal and social impact of living with recurrent UTIs.

D-mannose, a naturally occurring monosaccharide, has garnered interest due to its potential role in preventing UTIs by inhibiting bacterial adhesion to the urinary tract lining^4^. Given the limitations of conventional treatments like antibiotics, there is a growing need to explore alternative options for recurrent UTIs. Herein, we performed a systematic review and meta-analysis to evaluate whether d-mannose can be a reliable and effective preventive measure for recurrent UTIs.

## Methods

We conducted a systematic review and meta-analysis according to PRISMA (Preferred Reporting Items for Systematic Reviews and Meta-Analyses) guidelines and the Cochrane Handbook for Systematic Reviews of Interventions^5,6^. The protocol was prospectively registered at the OSF (Mutarelli, A; 2024, October 17. Efficacy and safety of D-mannose for recurrent urinary tract infections: A Systematic Review and Meta-analysis of RCTs. Retrieved from osf.io/wq2rt)^7^.

### Eligibility Criteria

The inclusion criteria for this meta-analysis were as follows: (1) randomized controlled trials (RCTs); (2) comparing D-mannose with control or antibiotics for the prevention of UTI in women; (3) with a minimum follow-up period of 3 months; and (4) reporting at least one relevant outcome. The exclusion criteria were: (1) case reports, commentaries, abstracts, editorials, letters, and reviews; (2) studies combining D-mannose with multiple interventions; and (3) studies lacking relevant populations or outcomes of interest.

### Search Strategy and Data Extraction

We systematically searched PubMed, Embase, and the Cochrane Library from inception to May 2024 using the following search terms: “d-mannose”, “urinary tract”, “infection, bacteriuria”, “tract infection”, “pyuria”, and “prophylaxis”. The full search string in each database is provided in the Supplement Material. Two investigators (AM and CEFV) independently screened titles, abstracts, and full texts to determine eligibility. Disagreements were resolved by consensus.

### Endpoints and Subgroups

The main outcome was the efficacy of D-mannose compared with a control or antibiotics in the prophylaxis of recurrent UTIs. The control group included patients receiving either a placebo or no treatment. A second outcome was the efficacy of D-mannose compared to a control in postmenopausal women.

### Quality Assessment

We assessed the quality and risk of bias in the included studies using the Cochrane Risk of Bias 2 tool (RoB-2) for randomized trials^6^. Two investigators (AM and CEFV) independently conducted the assessments, resolving disagreements through consensus.

### Statistical Analysis

All primary analyses adhered to the intention-to-treat principle. Binary outcomes were calculated using relative risks (RRs) with corresponding 95% confidence intervals (CIs), and p-values less than 0.05 were deemed significant for treatment effects. We applied the Mantel-Haenszel method for binary outcomes and the restricted maximum likelihood (REML) method as a variance estimator. All plots were generated using random-effects models.

Heterogeneity was assessed with Cochran’s Q test and the I^2^ statistics, with a p-value <0.10 and I^2^ ≥ 25% indicative of significant heterogeneity. Sensitivity analyses were performed using leave-one-out meta-analyses to evaluate each study’s influence on the overall effect estimate. All analyses were made using R version 4.2.1 (R Foundation for Statistical Computing, Vienna, Austria)^8^.

## Results

### Study Selection and Characteristics

**Figure 1:**
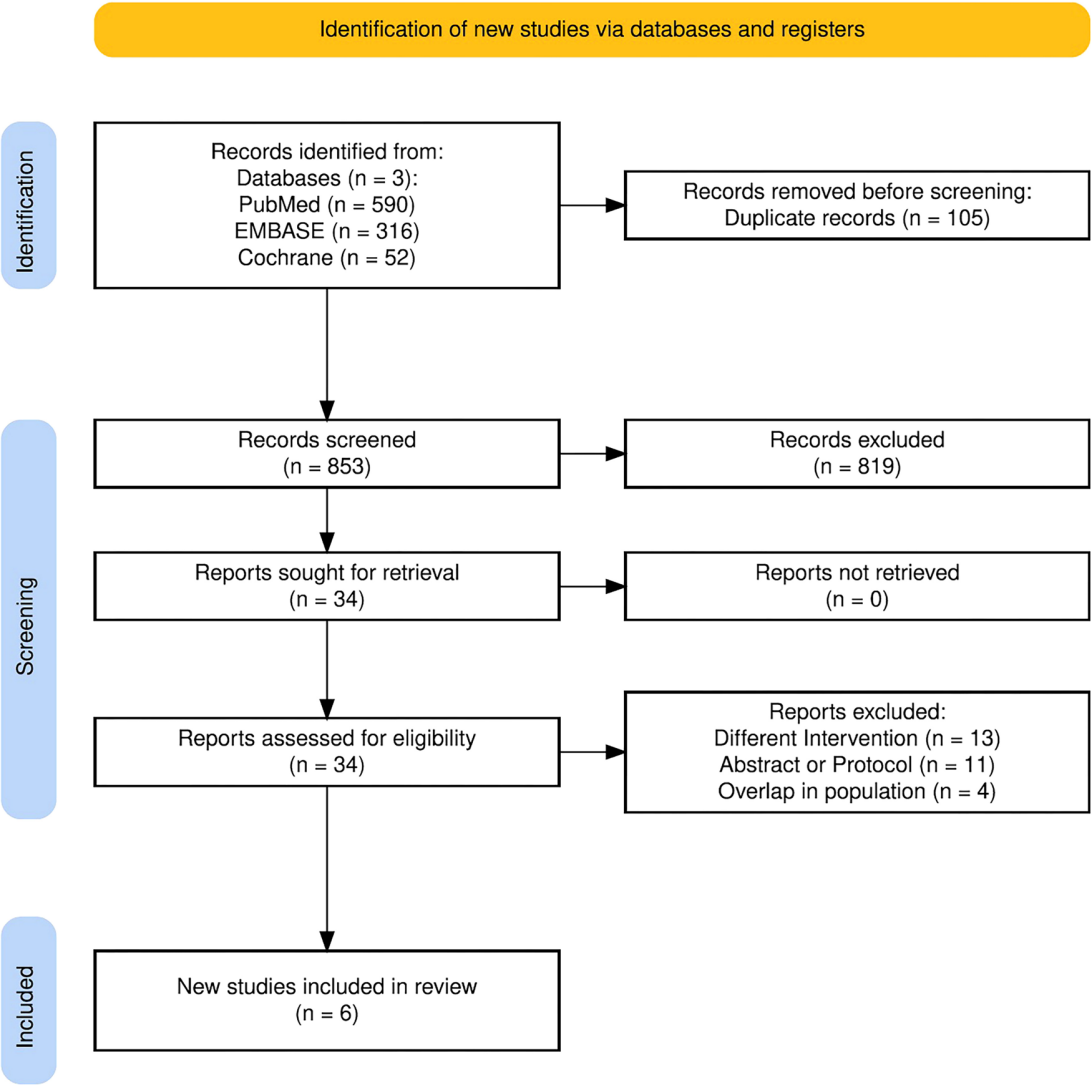
PRISMA flow chart of study selection.

Our search initially identified 958 papers, which were reduced to 853 after removing duplicates. Of these, 34 studies were selected for full-text review, and six RCTs were included in the analysis (Figure 1)^9,10,11,12,13,14^. Thirteen studies were excluded because they used different interventions, 11 were excluded for being conference abstracts or research protocols, and four were excluded due to population overlap. Among the six RCTs, two compared D-mannose with antibiotics, two compared it with placebo or no treatment, and two involved populations using either vaginal estrogen or proanthocyanidins. The largest study enrolled 598 participants, while the smallest had 43 participants.

A total of 1,167 participants were enrolled, 534 of whom received D-mannose. Most participants (98%, or 1,142 individuals) were women. Four studies had a six-month follow-up period, one had a four-month follow-up, and one had a three-month follow-up. Further details of study characteristics are available in Table 1.

**Table 1:** Characteristics of individual studies

Study	Follow Up	D-Mannose Dosage	Comparison	Sample		Age		Women	
				Intervention	Control	Age - intervention	Age - control	Women-intervention	Women-control
Hayward et al.^11^	6m	2g daily	Placebo	303	295	58.6 (17.1)	57.3 (19.1)	100%	100%
Domenici et al.^14^	6m	3g daily	No treatment	22	21	46.7 (5.7)		100%	100%
Kranjčec et al.^12^	6m	2g daily	Placebo vs Antibiotics	103 / 103	102	47.6 (13.5)	48.5 (13.5)	100%	100%
Porru et al.^13^	4m	2g daily	Antibiotics	60	60	39.2 (24.0)		100%	100%
Lenger et al.^10^	3m	2g daily	VET alone	19	25	–	–	100%	100%
Rau et al.^9^	6m	2g daily	PAC alone	27	27	59.9 (15.7)	59.1 (15.7)	52%	55%

### Efficacy of D-Mannose

**Figure 2:**
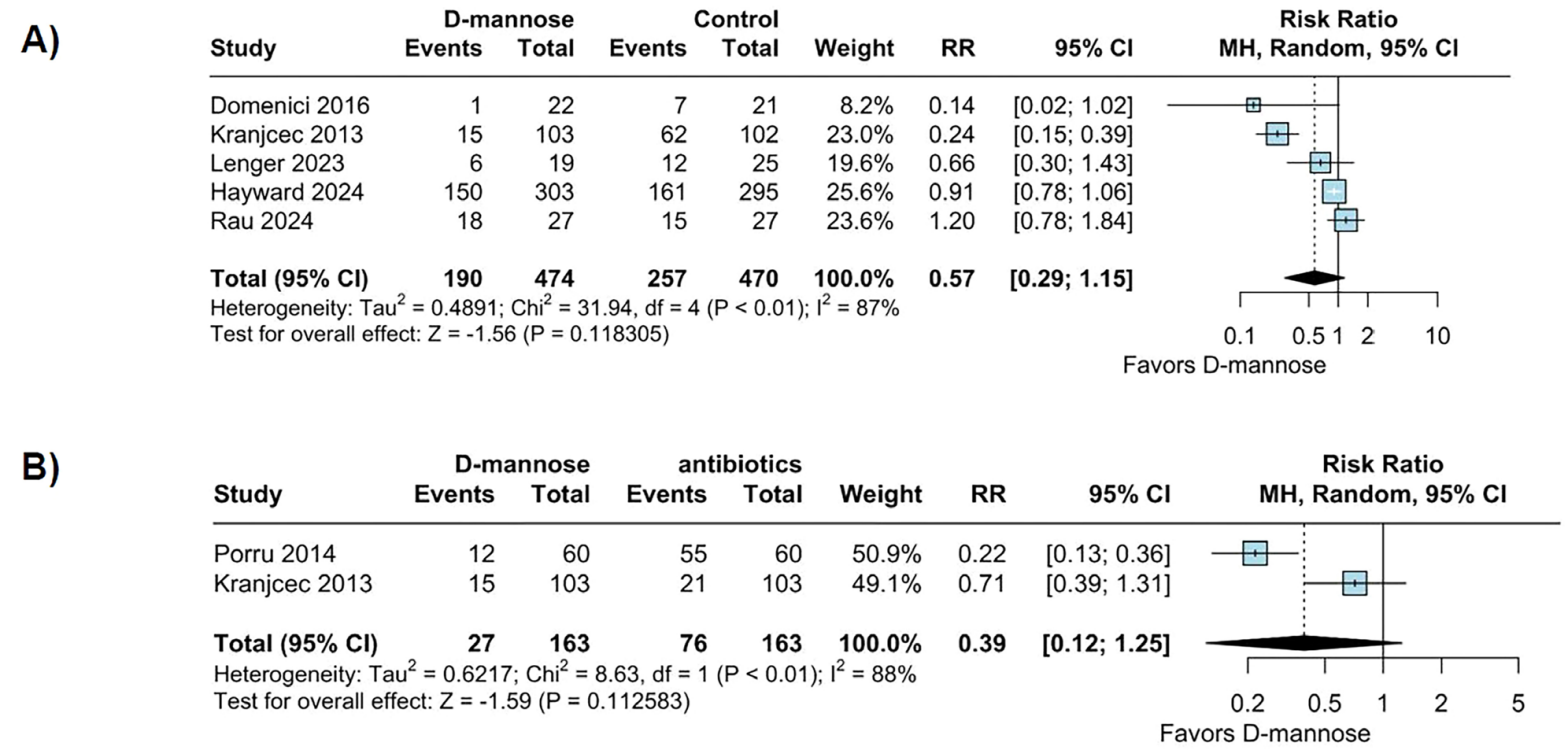
No difference was found comparing UTI recurrence between (A) D-mannose vs. control and (B) D-mannose vs. Antibiotics.

**Figure 3:**
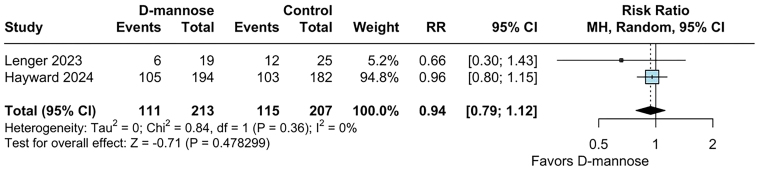
No difference was found between UTI recurrence and D-mannose vs. control in postmenopausal women.

In the pooled analysis, D-mannose was not associated with a significant reduction in UTI recurrence compared with control (RR 0.57; 95% CI 0.29 to 1.15; p = 0.118; I^2^ = 87%; Figure 2A) or antibiotics (RR 0.39; 95% CI 0.12 to 1.25; p = 0.13; I^2^ = 88%; Figure 2B). When analyzed specifically within the postmenopausal subgroup, D-mannose did not significantly lower the recurrence rate of UTIs (RR 0.94; 95% CI 0.79 to 1.12; p = 0.478; I^2^ = 0%; Figure 3).

### Sensitivity Analysis

**Figure 4:**
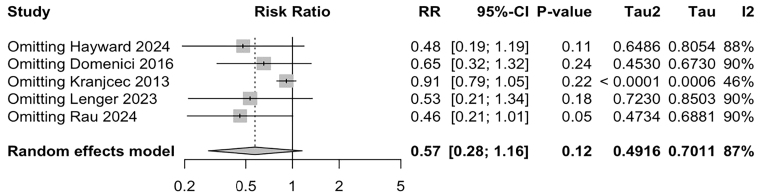
Leave-one-out of UTI recurrence comparing D-mannose vs Control with consistent findings.

To assess the robustness of our findings, we conducted a leave-one-out sensitivity analysis for the main outcome of UTI recurrence. This analysis confirmed the consistency of the results, with no single study significantly altering the overall efficacy estimate (Figure 4).

A Baujat plot identified the study by Kranjčec et al.^12^ as the largest contributor to the overall heterogeneity (Supplement Material Figure 1). Heterogeneity decreased from 87% to 46% when this study was excluded.

### Quality Assessment

The risk of bias was assessed using the Risk of Bias 2 (RoB 2) tool^6^. No study was found to have a high risk of bias (Supplement Material Figure 2). Only Domenici et al.^14^ raised some concerns in the first domain regarding the randomization process, as Table 1 in their article does not present the baseline characteristics for each group, instead it presents the combined data of both groups^14^. Furthermore, there is no clear description of the allocation procedure. A funnel plot was also generated to assess potential publication bias and small study effects, which showed asymmetry (Supplement Material Figure 3).

## Discussion

In this systematic review and meta-analysis of RCTs, we explored the efficacy of D-mannose for treating recurrent UTIs. Our analysis included six studies with a total of 1,167 participants, the majority of whom were women (1,144, 98%). Contrary to previous meta-analyses, we did not find any benefit in the use of D-mannose as prophylaxis for recurrent UTIs, whether compared with placebo or antibiotics^15^. No significant difference was found in the postmenopausal subgroup either. Our findings remained consistent in the leave-one-out sensitivity analyses.

D-mannose, an epimer of glucose, can be obtained from plants, fruits, and microorganisms using D-mannose isomerases^4^. It is theorized that D-mannose prevents bacterial adherence to uroepithelial cells^4,16,17^. Once ingested, it is rapidly absorbed and subsequently excreted in urine, reducing bacterial adhesion in the bladder^4,16,17^. By binding to bacteria, D-mannose prevents their attachment to uroepithelial cells, facilitating their elimination through urination^4,16,17^.

However, our analysis found no significant difference between D-mannose treatment and no treatment or placebo. Both Kranjčec and colleagues and Porru and colleagues reported a reduction in UTI recurrence in the intervention group^12,13^. Similarly, the previous meta-analysis also indicated a favorable outcome for the D-mannose group^15^. In contrast, Hayward et al.^11^, the largest trial comparing D-mannose with placebo, found no significant difference between D-mannose and control.

The main difference between these three studies is the mean age of participants and the sample size (Table 1)^11–13^. The two studies with positive outcomes had younger participants and smaller samples^12,13^. In addition, the follow-up time and D-mannose dosage were the same. In a sub-analysis by Hayward et al.^11^, premenopausal women treated with D-mannose had fewer recurrent UTIs, but no statistical test was conducted to confirm this finding. Future studies could explore the use of D-mannose in younger women.

It has been suggested that other non-antibiotic approaches may help prevent UTI recurrence^18,19^. Topical estrogen has been associated with reduced recurrence in postmenopausal women, while cranberry products have also shown potential in decreasing UTI recurrence^18,19^. However, these findings remain controversial by the existence of contrasting evidence^20^. As such, the most reliable approach to managing UTI recurrence remains the use of antibiotics^21^.

Our study has limitations. First, the number of available studies is limited, and they present substantial heterogeneity, contributing to overall heterogeneity in the meta-analysis. However, our findings remained consistent in the leave-one-out sensitivity analysis. Second, only one double-blind RCT was included in the analyis^11^. Third, few studies conducted subgroup analyses, such as for postmenopausal women, resulting in limited data to assess specific subgroups that might benefit from D-mannose treatment. Finally, we did not have access to individual patient data, which prevented us from conducting a more granular subanalysis according to age, comorbidities, or antibiotic use.

## Conclusion

Our meta-analysis evaluating D-mannose for the prevention of recurrent UTI found no significant difference between the intervention and control groups. Further studies focusing on specific subgroups may help clarify the potential benefits of D-mannose, particularly with improved designs such as double-blinded randomized controlled trials.

## Data Availability

Data are available within reasonable request.

## Supplementary Material

The following online material is available for this article:

Table S1 – Search strategies.

Figure S1 – Baujat plot.

Figura S2 – Risk of Bias RoB2.

Figure S3 – Funnel plot.
