# Supplementary material for: Efficacy of D-mannose as prophylaxis of recurrent urinary tract infection: a systematic review and meta-analysis of randomized controlled trials
Source: J Bras Nefrol. 2025 Sep 26;47(4):e20250169. doi: 10.1590/2175-8239-JBN-2025-0169en (PMC12471090; doi:10.1590/2175-8239-JBN-2025-0169en)
Supplement: Table S1 [file 2175-8239-jbn-47-4-e20250169-supp1.pdf]

Braz. J. Nephrol.
https://doi.org/10.1590/2175-8239-JBN-2025-0169en

Supplementary Material to "Efficacy of D-mannose as prophylaxis of recurrent urinary tract infection: a systematic review and meta-analysis of randomized controlled trials"

Table S1 - Search strategy.

Data base	Search strategy
**EMBASE**	D-mannose AND ("urinary tract" OR infection OR bacteriuria OR "tract infection" OR pyuria) AND (prophylaxis OR treatment OR therapy OR prevent OR control)
**PubMed**	D-mannose AND ("urinary tract" OR infection OR bacteriuria OR "tract infection" OR pyuria) AND (prophylaxis OR treatment OR therapy OR prevent OR control) AND ("randomized controlled trial"[pt] OR "controlled clinical trial"[pt] OR randomized[tiab] OR placebo[tiab] OR "drug therapy"[sh] OR randomly[tiab] OR trial[tiab] OR groups[tiab]) NOT ("Animals"[Mesh] NOT "Humans"[Mesh])
**Cochrane**	D-mannose AND ("urinary tract" OR infection OR bacteriuria OR "tract infection" OR pyuria) AND (prophylaxis OR treatment OR therapy OR prevent OR control) AND ("randomized controlled trial"[pt] OR "controlled clinical trial"[pt] OR randomized[tiab] OR placebo[tiab] OR "drug therapy"[sh] OR randomly[tiab] OR trial[tiab] OR groups[tiab]) NOT ("Animals"[Mesh] NOT "Humans"[Mesh])
