# Supplementary material for: Efficacy of D-mannose as prophylaxis of recurrent urinary tract infection: a systematic review and meta-analysis of randomized controlled trials
Source: J Bras Nefrol. 2025 Sep 26;47(4):e20250169. doi: 10.1590/2175-8239-JBN-2025-0169en (PMC12471090; doi:10.1590/2175-8239-JBN-2025-0169en)
Supplement: Figure S1 [file 2175-8239-jbn-47-4-e20250169-supp2.pdf]

Braz. J. Nephrol.
https://doi.org/10.1590/2175-8239-JBN-2025-0169en

Supplementary Material to "Efficacy of D-mannose as prophylaxis of recurrent urinary tract infection: a systematic review and meta-analysis of randomized controlled trials"

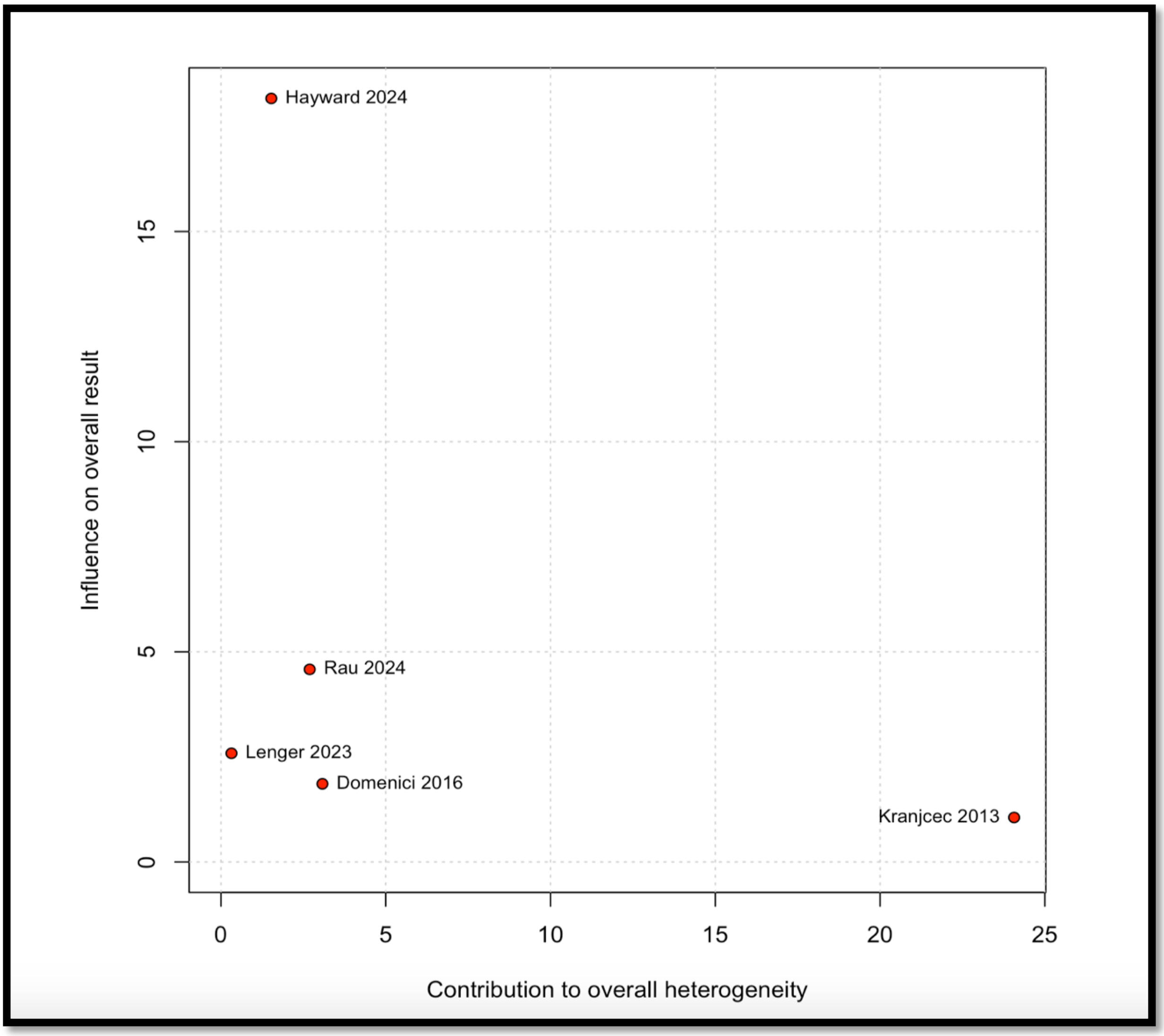

Figure S1 - Baujat plot – Influence on overall result and the contribution of heterogeneity.
