# Supplementary material for: Efficacy of D-mannose as prophylaxis of recurrent urinary tract infection: a systematic review and meta-analysis of randomized controlled trials
Source: J Bras Nefrol. 2025 Sep 26;47(4):e20250169. doi: 10.1590/2175-8239-JBN-2025-0169en (PMC12471090; doi:10.1590/2175-8239-JBN-2025-0169en)
Supplement: Translation

# Translation: Eficácia da D-manose como profilaxia da infecção recorrente do trato urinário: uma revisão sistemática e metanálise de ensaios clínicos randomizados controlados

**DOI:** 10.1590/2175-8239-JBN-2025-0169pt

**Authors:** Carlos Eduardo Franca Vargas, Antonio Mutarelli, Luis Gustavo Menegardo, Acza Kalica Buarque da Silva, Patricia Rocha Barros Vieira, Jiandra da Luz, Nicole Felix, Luis Claudio Santos Pinto

**Affiliations:** 1 Universidade Federal de Minas Gerais, Faculdade de Medicina, Belo Horizonte, MG, Brasil.; 2 Massachusetts General Hospital, Cardiovascular Research Center, Boston, EUA.; 3 Escola Superior de Ciência da Santa Casa de Misericórdia de Vitória, Vitória, ES, Brasil.; 4 Universidade Federal de Alagoas, Faculdade de Medicina, Maceió, AL, Brasil.; 5 Universidade Estadual de Campinas, Campinas, SP, Brasil.; 6 Universidad Nacional de La Plata, La Plata, Argentina.; 7 Universidade Federal de Campina Grande, Campina Grande, PB, Brasil.; 8 Centro Universitário Metropolitano da Amazônia, Belém, PA, Brasil.

**Keywords:** Manose, Antibioticoprofilaxia, Infecções Urinárias, Revisão Sistemática, Metanálise

## Abstract

**Introdução:**

As infecções do trato urinário (ITUs) recorrentes afetam significativamente a qualidade de vida devido aos sintomas, aos efeitos sobre a atividade sexual, à dor persistente e ao uso recorrente de antibióticos. Esta revisão sistemática e metanálise teve como objetivo avaliar a eficácia da D-manose na prevenção de ITUs recorrentes.

**Métodos:**

Em maio de 2024, realizamos uma busca sistemática nas bases de dados PubMed, EMBASE e *Cochrane Library* por ensaios clínicos randomizados (ECRs) que comparassem o tratamento com D-manose à ausência de intervenção ou à terapia antibiótica padrão em pacientes com alto risco de ITU recorrente. Aplicamos um modelo de efeitos aleatórios para agrupar os riscos relativos (RR) e os intervalos de confiança (IC) de 95%.

**Resultados:**

Incluímos 6 ECRs envolvendo 1.167 participantes, dos quais 534 receberam D-manose e 521 (97,6%) eram mulheres. A D-manose não se associou a uma redução na ITU recorrente em comparação ao grupo controle (RR: 0,57; IC 95% 0,29 – 1,15; p < 0,01) ou ao uso de antibióticos (RR: 0,39; IC 95% 0,12 – 1,25; p < 0,01). Análises adicionais demonstraram que a D-manose não melhorou os desfechos em um subgrupo de mulheres na pós-menopausa.

**Conclusão:**

Nesta metanálise de ECRs, a D-manose não reduziu a incidência de ITUs recorrentes em comparação ao grupo controle ou ao tratamento com antibióticos em pacientes de alto risco.

## Introdução

Em 2019, mais de 400 milhões de pacientes apresentaram infecções do trato urinário (ITUs), e mais de 200 mil foram a óbito em decorrência da doença^1^. A maioria dos pacientes é do sexo feminino e frequentemente apresenta ITUs recorrentes, com mais de dois episódios em seis meses ou três em um ano^2^. As ITUs têm um impacto negativo na qualidade de vida e podem causar dor e levar à evitação de relações sexuais.

Embora os antibióticos permaneçam como o tratamento principal, sua eficácia pode variar, e o uso prolongado levanta preocupações sobre resistência e efeitos adversos^2^. Algumas mulheres se beneficiam de um atendimento médico empático e bem informado, que pode contribuir para aliviar os sintomas, mas muitas continuam a enfrentar dificuldades, apesar das intervenções médicas^3^. Isso destaca a necessidade de pesquisas mais amplas sobre tratamentos alternativos, incluindo opções não antibióticas, como probióticos ou remédios naturais, e uma compreensão mais profunda do impacto pessoal e social de conviver com ITUs recorrentes.

A D-manose, um monossacarídeo natural, tem despertado interesse devido ao seu potencial papel na prevenção de ITUs, ao inibir a adesão bacteriana ao epitélio urinário^4^. Dadas as limitações dos tratamentos convencionais, como os antibióticos, há uma necessidade crescente de se explorar opções alternativas para ITUs recorrentes. Neste artigo, realizamos uma revisão sistemática e metanálise para avaliar se a D-manose pode ser uma medida preventiva confiável e eficaz nesse contexto.

## Métodos

Realizamos uma revisão sistemática e metanálise de acordo com as diretrizes PRISMA (*Preferred Reporting Items for Systematic Reviews and Meta-Analyses*) e com o *Cochrane Handbook for Systematic Reviews of Interventions* ^5,6^. O protocolo foi registrado prospectivamente na plataforma OSF (Mutarelli, A; 2024, 17 de outubro. *Efficacy and safety of D-mannose for recurrent urinary tract infections: A Systematic Review and Meta-analysis of RCTs*. Retirado de osf.io/wq2rt)^7^.

### Critérios de Elegibilidade

Os critérios de inclusão para esta metanálise foram os seguintes: (1) ensaios clínicos randomizados (ECRs); (2) que comparassem a D-manose com controles ou antibióticos para a prevenção de ITUs em mulheres; (3) com um período mínimo de acompanhamento de 3 meses; e (4) que relatassem ao menos um desfecho relevante. Os critérios de exclusão foram: (1) relatos de casos, comentários, resumos, editoriais, cartas e revisões; (2) estudos que combinaram D-manose com múltiplas intervenções; e (3) estudos sem populações relevantes ou desfechos de interesse.

### Estratégia de Busca e Extração de Dados

Realizamos uma busca sistemática nas bases de dados PubMed, Embase e *Cochrane Library*, desde o início até maio de 2024, utilizando os seguintes termos de pesquisa: “d-manose”, “trato urinário”, “infecção, bacteriúria”, “infecção do trato”, “piúria” e “profilaxia”. A sequência de pesquisa completa em cada banco de dados está disponível no Material Suplementar. Dois pesquisadores (AM e CEFV) realizaram, de forma independente, a triagem dos títulos, resumos e textos completos para determinar a elegibilidade dos estudos. As divergências foram resolvidas por consenso.

### Desfechos e Subgrupos

O principal desfecho foi a eficácia da D-manose, em comparação com um controle ou com antibióticos, na profilaxia de ITUs recorrentes. O grupo controle incluiu pacientes que receberam placebo ou nenhum tratamento. Um segundo desfecho foi a eficácia da D-manose, em comparação com um grupo controle de mulheres na pós-menopausa.

### Avaliação de Qualidade

A qualidade e o risco de viés dos estudos incluídos foram avaliados por meio da ferramenta *Cochrane Risk of Bias 2* (RoB-2) para ensaios clínicos randomizados^6^. Dois pesquisadores (AM e CEFV) realizaram as avaliações de forma independente, resolvendo divergências por meio de consenso.

### Análise Estatística

Todas as análises primárias seguiram o princípio da análise por intenção de tratar. Os desfechos binários foram calculados utilizando riscos relativos (RRs) com intervalos de confiança (ICs) de 95% correspondentes, com valores de p inferiores a 0,05 considerados significativos para os efeitos do tratamento. Aplicamos o método de Mantel-Haenszel para desfechos binários e o método de máxima verossimilhança restrita (REML) como estimador de variância. Todos os gráficos foram gerados a partir de modelos de efeitos aleatórios.

A heterogeneidade foi avaliada por meio do teste Q de Cochran e da estatística I^2^, com um valor de p < 0,10 e I^2^ ≥ 25% como indicativos de heterogeneidade significativa. Foram realizadas análises de sensibilidade utilizando o método *leave-one-out* para avaliar a influência de cada estudo na estimativa total do efeito. Todas as análises foram realizadas utilizando o R versão 4.2.1 (*R Foundation for Statistical Computing*, Viena, Áustria)^8^.

## Resultados

### Seleção e Características do Estudo

Nossa pesquisa identificou inicialmente 958 artigos, os quais foram reduzidos para 853 após a remoção de duplicatas. Destes, 34 estudos foram selecionados para revisão completa do texto, e seis ECRs foram incluídos na análise (Figura 1)^9,10,11,12,13,14^. Treze estudos foram excluídos por utilizarem intervenções diferentes, 11 foram excluídos por serem resumos de congressos ou protocolos de pesquisa e quatro foram excluídos devido à sobreposição de populações. Entre os seis ECRs, dois compararam a D-manose com antibióticos, dois a compararam com placebo ou ausência de tratamento e dois envolveram populações que faziam uso de estrogênio vaginal ou proantocianidinas. O maior estudo envolveu 598 participantes, enquanto o menor contou com 43 participantes.

**Figura 1:** Fluxograma PRISMA da seleção dos estudos.

Um total de 1.167 participantes foram inscritos, dos quais 534 receberam D-manose. A maioria dos participantes (98%, ou 1.142 indivíduos) eram mulheres. Quatro estudos apresentaram um período de acompanhamento de seis meses, um teve um acompanhamento de quatro meses e outro, de três meses. Detalhes adicionais sobre as características dos estudos estão disponíveis na Tabela 1.

**Tabela 1:** Características dos Estudos Individuais

Estudo	Acompanhamento	Dosagem de D-Manose	Comparação	Amostra		Idade		Mulheres	
				Intervenção	Controle	Idade - intervenção	Idade - controle	Mulheres-intervenção	Mulheres-controle
Hayward et al.^11^	6m	2g por dia	Placebo	303	295	58,6 (17,1)	57,3 (19,1)	100%	100%
Domenici et al.^14^	6m	3g por dia	Sem tratamento	22	21	46,7 (5,7)		100%	100%
Kranjčec et al.^12^	6m	2g por dia	Placebo vs. Antibióticos	103 / 103	102	47,6 (13,5)	48,5 (13,5)	100%	100%
Porru et al.^13^	4m	2g por dia	Antibióticos	60	60	39,2 (24,0)		100%	100%
Lenger et al.^10^	3m	2g por dia	Estrogênio vaginal isolado	19	25	–	–	100%	100%
Rau et al.^9^	6m	2g por dia	Proantocianidinas isoladas	27	27	59,9 (15,7)	59,1 (15,7)	52%	55%

### Eficácia da D-Manose

Na análise combinada, a D-manose não foi associada a uma redução significativa na recorrência de ITUs em comparação ao controle (RR 0,57; IC 95% 0,29 a 1,15; p = 0,118; I^2^ = 87%; Figura 2A) ou aos antibióticos (RR 0,39; IC 95% 0,12 a 1,25; p = 0,13; I^2^ = 88%; Figura 2B). Quando analisada especificamente no subgrupo pós-menopausa, a D-manose não reduziu significativamente a taxa de recorrência de ITUs (RR 0,94; IC 95% 0,79 a 1,12; p = 0,478; I^2^ = 0%; Figura 3).

**Figura 2:** Não foram observadas diferenças na recorrência de ITUs ao comparar (A) D-manose vs. controle e (B) D-manose vs. antibióticos.

**Figura 3:** Não foram observadas diferenças entre a recorrência de ITUs e a D-manose vs. controle em mulheres na pós-menopausa.

### Análise de Sensibilidade

Para avaliar a robustez de nossos achados, realizamos uma análise de sensibilidade *leave-one-out* para o desfecho principal de recorrência de ITU. Essa análise confirmou a consistência dos resultados, sem que nenhum estudo alterasse significativamente a estimativa geral de eficácia (Figura 4).

**Figura 4:** Análise leave-one-out da recorrência de ITUs comparando D-manose vs. controle com achados consistentes.

O gráfico de Baujat identificou o estudo de Kranjčec et al.^12^ como o maior contribuinte para a heterogeneidade global (Figura Material Suplementar 1). A heterogeneidade diminuiu de 87% para 46% quando este estudo foi excluído.

### Avaliação de Qualidade

O risco de viés foi avaliado utilizando a ferramenta *Risk of Bias 2* (RoB 2)^6^. Nenhum estudo apresentou alto risco de viés ( Figura Material Suplementar 2). Apenas Domenici et al.^14^ levantaram algumas preocupações no primeiro domínio, no que diz respeito ao processo de randomização, uma vez que a Tabela 1 de seu artigo não apresenta as características basais de cada grupo, apresentando, em vez disso, os dados combinados de ambos os grupos^14^. Além disso, não há uma descrição clara do procedimento de alocação. Também foi gerado um gráfico de funil para avaliar o potencial viés de publicação e os efeitos de estudos de pequeno porte, o qual apresentou assimetria (Figura Material Suplementar 3).

## Discussão

Nesta revisão sistemática e metanálise de ECRs, investigamos a eficácia da D-manose no tratamento de ITUs recorrentes. Nossa análise incluiu seis estudos com um total de 1.167 participantes, a maioria mulheres (1.144; 98%). Em contraste com metanálises anteriores, não encontramos nenhum benefício no uso da D-manose como profilaxia para ITUs recorrentes, seja em comparação com placebo ou antibióticos^15^. Também não foi observada diferença significativa no subgrupo pós-menopausa. Nossos achados se mantiveram consistentes nas análises de sensibilidade *leave-one-out*.

A D-manose, um epímero da glicose, pode ser obtida a partir de plantas, frutas e microrganismos utilizando isomerases de D-manose^4^. Teoriza-se que a D-manose impede a adesão bacteriana às células uroepiteliais^4,16,17^. Uma vez ingerida, é rapidamente absorvida e posteriormente excretada na urina, reduzindo a adesão bacteriana na bexiga^4,16,17^. Ao ligar-se às bactérias, a D-manose impede a sua fixação às células uroepiteliais, facilitando sua eliminação por meio da micção^4,16,17^.

No entanto, nossa análise não encontrou diferença significativa entre o tratamento com D-manose e a ausência de tratamento ou o uso de placebo. Tanto Kranjčec e colegas quanto Porru e colegas relataram uma redução na recorrência de ITUs no grupo de intervenção^12,13^. De forma semelhante, a metanálise anterior também indicou um desfecho favorável para o grupo tratado com D-manose^15^. Em contrapartida, Hayward et al.^11^, o maior ensaio que comparou D-manose com placebo, não encontrou diferença significativa entre D-manose e controle.

A principal diferença entre esses três estudos está na média de idade dos participantes e no tamanho da amostra (Tabela 1)^11–13^. Os dois estudos com desfechos positivos envolveram participantes mais jovens e amostras menores^12,13^. Além disso, o tempo de acompanhamento e a dosagem de D-manose foram os mesmos. Em uma subanálise realizada por Hayward et al.^11^, mulheres na pré-menopausa tratadas com D-manose apresentaram menos episódios recorrentes de ITUs, mas nenhum teste estatístico foi realizado para confirmar esse achado. Estudos futuros poderiam explorar o uso da D-manose em mulheres mais jovens.

Tem sido sugerido que outras abordagens não antibióticas podem auxiliar na prevenção da recorrência da ITU^18,19^. O estrogênio tópico tem sido associado à redução da recorrência em mulheres na pós-menopausa, enquanto produtos à base de *cranberry* também demonstraram potencial na redução da recorrência de ITUs^18,19^. No entanto, esses achados permanecem controversos devido à existência de evidências conflitantes^20^. Assim, a abordagem mais confiável para o manejo da recorrência da ITU continua sendo o uso de antibióticos^21^.

Nosso estudo apresenta limitações. Primeiramente, o número de estudos disponíveis é limitado e apresenta heterogeneidade substancial, contribuindo para a heterogeneidade geral da metanálise. No entanto, nossos achados permaneceram consistentes na análise de sensibilidade *leave-one-out*. Em segundo lugar, apenas um ECR duplo-cego foi incluído na análise^11^. Terceiro, poucos estudos realizaram análises de subgrupos, como mulheres na pós-menopausa, resultando em dados limitados para avaliar subgrupos específicos que poderiam se beneficiar do tratamento com D-manose. Por fim, não tivemos acesso aos dados individuais dos pacientes, o que impossibilitou a realização de uma subanálise mais detalhada com base na idade, comorbidades ou uso de antibióticos.

## Conclusão

Nossa metanálise, que avaliou o uso da D-manose na prevenção de ITUs recorrentes, não encontrou diferenças significativas entre os grupos de intervenção e controle. Estudos adicionais, com foco em subgrupos específicos, podem ajudar a esclarecer os potenciais benefícios da D-manose, especialmente se forem realizados com desenhos melhorados, como ensaios clínicos randomizados duplo-cegos.

## Data Availability

Os dados estão disponíveis mediante solicitação razoável.

## Material Suplementar

O seguinte material on-line está disponível para este artigo:

Tabela S1 – Estratégias de busca.

Figura S1 – Gráfico de Baujat.

Figura S2 – Risco de viés (RoB2).

Figura S3 – Gráfico de funil.
